# The road from PhD in Germany to postdoc in the USA

**DOI:** 10.1002/ansa.202200041

**Published:** 2022-12-08

**Authors:** Caroline Knittel

**Affiliations:** ^1^ Department of Chemistry and Biochemistry University of California San Diego La Jolla California USA

Close to the end of their PhD many young researchers are confronted with the question of whether to apply for a postdoctoral position or directly for jobs in industry. While starting a career in industry shortly after finishing a PhD degree can help to achieve high‐ranking positions earlier, extending the academic education in form of a postdoc can bring many advantages for future career goals in academia as well as in industry.^1^ However, the decision whether to seek a postdoctoral position should be well conceived. A previous study suggests that ten years after accomplishing a PhD the average ex‐postdoc salary is lower than for non‐postdocs.^2^ Nevertheless, this study was conducted solely for biomedical studies in the United States and might be different in other countries and for other research fields. Furthermore, a postdoc experience is not only useful to learn new skill sets in a foreign research environment but can also help to acquire a great set of soft skills.

As a current German postdoc in the United States, I would like to outline several factors that can lead to a positive US postdoc experience. First, it is important to start to plan early before the end of the PhD since finding the right group as well as the bureaucratic process can take a lot of time. I recommend planning at least 1 year in advance. The most difficult decision is probably which research institute and group to apply to. In this context, I agree with previous studies that underline the importance of the research environment, collaborations, and quality of supervision for beneficial postdoctoral training.^1^ Therefore, the receiving research institute and group needs to be selected with care and it can be helpful to inquire whether the department has a great research network with other research groups or has engaged in various collaborations in the past. Furthermore, it can also be beneficial to talk to other group members before accepting a postdoctoral position. They might give valuable insights not only into the quality of the supervision by the principal investigator but also into how well the knowledge transfer within the group has been established.

One of the most important aspects is financial funding. While several research groups might have fully funded postdoctoral positions available, others might require a fully sponsored fellowship from international organizations such as EMBO, HFSB, or national organizations such as the German research foundation (Deutsche Forschungsgemeinschaft) for Germany‐based candidates. The fellowship application process can take between 3 and 9 months and requires a lot of effort, particularly during the end of a PhD.

After the successful application and acceptance of a postdoctoral position, the J1‐visa application and interview process can take up to several months depending on the location and workload of the embassy. First, the postdoctoral candidate needs to receive the offer letter and DS‐2019 document from the receiving institute which is necessary to initiate the visa application. Afterward, the candidate needs to electronically fill out the DS‐160 form via ustraveldocs.com (not the embassy's website), pay an application fee, and schedule an appointment for the visa interview. The wait times for the visa interview can take weeks or even months. However, wait times vary between the different locations of the embassies. Therefore, it is worthwhile checking the appointment availability of an embassy in a different city on the ustraveldocs website when the wait time for the nearest embassy is too long.

Furthermore, every postdoc is required to have health insurance during the entire duration of the program. Health insurance can be obtained either in the home country through the fellowship organization and from private insurance companies, or directly through the receiving institute. Many postdocs choose to be insured directly through the receiving institute to avoid first advancing money for expensive medical bills and then filing for reimbursement in the home country.

Many married researchers who seek a postdoctoral position in the United States think it implies a long separation time from their spouses. However, the spouse, as well as children, can accompany the postdoc by applying for a J‐2 visa. Once arrived in the United States, the spouse can also apply for an employment authorization document that allows them to legally seek work during the time of the postdoc program of the J1 visa holder. This fact constitutes a great chance for the whole family of the future postdoc to thrive from a positive postdoc experience abroad.

Finally, housing needs to be chosen very carefully. While most rental companies offer virtual tours of their rental apartments, I do not recommend signing a lease without seeing the apartment in person. These remote tours often look a lot better on screen than in reality and other factors such as noise or the quality of the neighborhood are not transmitted virtually. Therefore, it can be very frustrating for a postdoc when a lease was signed remotely for an entire year, but the actual living situation turns out to be unsatisfactory. As an alternative, the future postdoc can book a short‐term room or apartment for the first weeks of the appointment and go to apartment visits in person after arrival at the destination. This does not only reflect the actual housing quality but also helps to avoid possible scams.

Ultimately, the overall postdoc application process is long (6–12 months in total) and laborious. However, a postdoc can be positive and rewarding when well‐planned which I can confirm due to my own positive experience. While it can be daunting for many young researchers to spend additional years in academia and earn a lower salary in the short term than in post PhD industry jobs, a postdoc can be highly valuable for the personal development of a scientist and lead into a new direction for the future career.
